# Rapidly reversible total facial paralysis after lipofilling around facial nerve truncus

**DOI:** 10.1080/23320885.2026.2724721

**Published:** 2026-08-31

**Authors:** Ersin Yavuz, Emre Berk Bulut, Alp Ercan

**Affiliations:** a Department of Plastic, Reconstructive and Aesthetic Surgery, Diyarbakır Gazi Yaşargil Training and Research Hospital, Diyarbakır, Turkey; b Department of Plastic, Reconstructive and Aesthetic Surgery, Istanbul University-Cerrahpaşa, Istanbul, Turkey

**Keywords:** Facial nerve paralysis, lipofilling, radiotherapy, Anterolateral thigh flap, Neuropraxia

## Abstract

Autologous fat grafting is widely used for secondary contour refinement following head and neck reconstruction and is generally considered safe. However, irradiated reconstructed tissues exhibit fibrosis and reduced compliance, potentially predisposing patients to uncommon complications. Transient facial nerve paralysis following fat grafting has not previously been reported in an irradiated anterolateral thigh (ALT) free flap. A 57-year-old man underwent extensive oncologic resection for verrucous squamous cell carcinoma involving the temporal bone and parotid region, followed by ALT free flap reconstruction and adjuvant radiotherapy. After flap debulking and an initial lipofilling procedure, a second fat-grafting session (60 mL) was performed five months later for residual preauricular contour deformity. Immediately after surgery, the patient developed House–Brackmann grade V facial paralysis. Systemic methylprednisolone was initiated at 64 mg/day and tapered over five days. Facial function improved rapidly, with complete recovery within 36 hours and no residual deficit. Given the close temporal relationship to fat grafting and rapid recovery, transient pressure-related neuropraxia within the irradiated fibrotic tissue bed was considered a plausible mechanism; however, this remains hypothetical without objective evidence of nerve compression, edema, or ischemia. To our knowledge, this is the first reported case of rapidly reversible total facial paralysis following autologous fat grafting in an irradiated ALT free flap. Reduced tissue compliance secondary to radiation-induced fibrosis may contribute to transient nerve dysfunction. Awareness of this potential complication and cautious, low-pressure injection may be important when performing fat grafting near major nerve structures in irradiated reconstructive fields.

## 1. Introduction

Facial reconstruction following extensive oncologic resection remains challenging, particularly in defects involving the temporal bone and parotid region. The Anterolateral Thigh (ALT) Flap is frequently used for these defects because of its reliable vascular pedicle and large tissue volume [1]. However, postoperative radiotherapy (RT) and subsequent tissue atrophy may compromise long-term aesthetic and functional outcomes, often requiring secondary refinement procedures such as lipofilling [2].

Although autologous fat grafting is generally considered safe for volumetric restoration, its use in previously irradiated and reconstructed tissues may present unique risks. Altered tissue compliance and microvascular changes in irradiated beds can lead to unexpected complications during or after injection. While acute facial nerve dysfunction has been reported after synthetic dermal filler injection, transient total paralysis specifically triggered by autologous fat grafting in an irradiated free flap has not been reported to date.

The aim of this study is to present the first known case of sudden-onset total facial nerve trunk paralysis after fat grafting into an irradiated ALT flap, its rapid clinical recovery, and a possible pressure-related pathophysiological mechanism.

## 2. Patients/material and methods

A 57-year-old male presented with a tumor infiltrating the mastoid prominence and extending to the temporomandibular joint. He underwent extensive surgical resection including mastoidectomy, stapedectomy (removal of the malleus and incus), middle ear mucosal resection, mandibular condylectomy, total parotidectomy, partial ear amputation, and modified radical neck dissection. Reconstruction was performed using an Anterolateral Thigh (ALT) Flap. Histopathology confirmed verrucous Squamous Cell Carcinoma with a close margin at the middle ear, while peripheral margins were negative.

**Figure 1.**
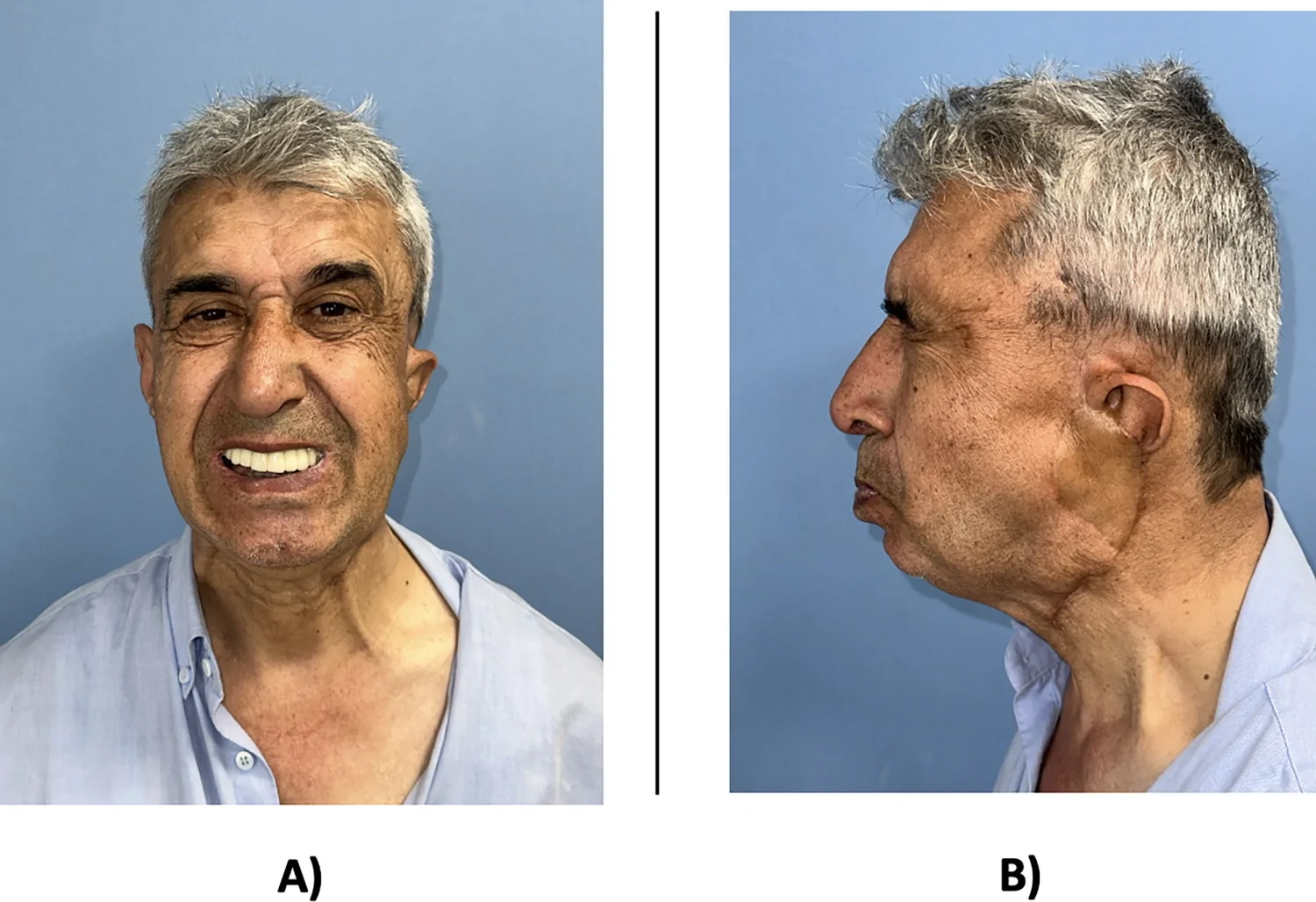
Long-term postoperative view before the complication. (A) Anteroposterior clinical view of the patient one year postoperatively, showing normal facial animation and symmetric nerve function. (B) Lateral view of the same patient, highlighting the volumetric atrophy in the servico-facial transition zone. *This atrophy was the target for the subsequent fat grafting that led to acute facial paralysis.*

Following surgery, the patient completed 30 sessions of adjuvant radiotherapy (RT). Five months later, surgical flap thinning (debulking) was performed to reduce neck bulkiness and improve contour **(**Figure 1A and B). During the same procedure, autologous fat grafting was performed to correct the residual preauricular volume deficiency. The abdominal donor site was infiltrated with standard Klein solution, and 20 cc of adipose tissue was harvested. The harvested fat was mechanically processed by passing it between syringes 20 times to obtain a more refined graft consistency. The processed fat was then injected predominantly into the deep adipose layer of the ALT flap and the immediately underlying subflap plane. Injection was performed slowly and with minimal pressure to avoid excessive local tissue tension. No intraoperative or early postoperative complications occurred.

**Figure 2.**
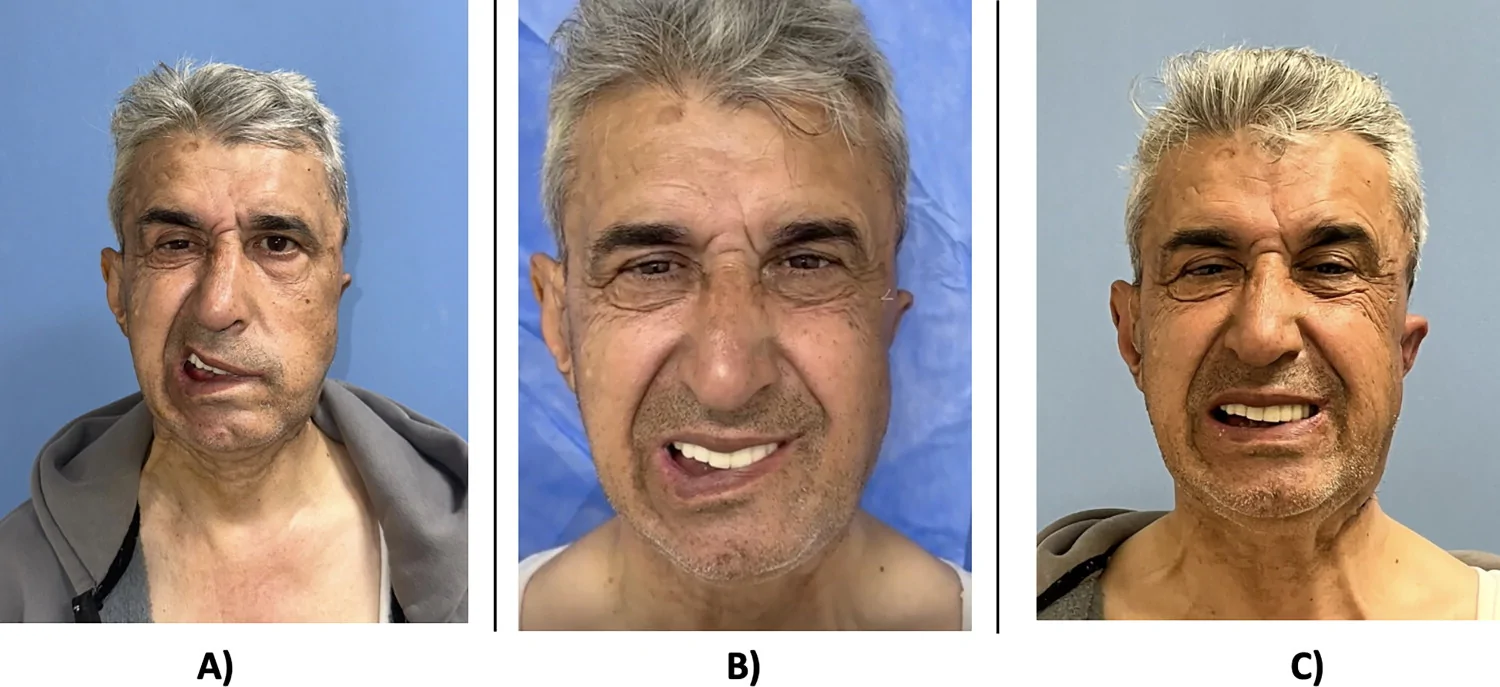
Clinical progression and response to systemic corticosteroid therapy. (A) Immediate postoperative view following the second lipofilling session, demonstrating sudden-onset left-sided facial nerve paralysis (House–Brackmann Grade V). (B) Partial recovery of facial animation observed after initiation of systemic methylprednisolone therapy. (C) Complete functional restoration of the facial nerve observed within 36 h after the onset of paralysis.

A second lipofilling session was performed 5 months later using the same harvesting, processing, and low-pressure injection technique. During this session, 60 cc of processed fat graft was distributed within the same deep intraflap and subflap tissue planes to further correct the residual contour deficiency. Immediately after surgery, the patient developed sudden-onset facial paralysis. Suspecting acute mechanical compression secondary to edema affecting the facial nerve branches beneath the deep surface of the irradiated fibrotic ALT flap, systemic methylprednisolone therapy was initiated at 64 mg/day. The dose was reduced by half each day (64, 32, 16, 8, and 4 mg/day) and discontinued after a total treatment duration of 5 days. The patient had no known comorbidities, including diabetes mellitus. Capillary blood glucose levels were monitored twice daily throughout corticosteroid therapy, and no clinically significant hyperglycemia or other disturbance in glycemic control was observed. Clinical improvement began rapidly and complete facial nerve recovery was achieved within 36 h (Figure 2A–C). The patient was discharged after one week with full restoration of function. Written informed consent was obtained from the patient for participation and for publication of the clinical information and images included in this case report.

## 3. Discussion

Facial paralysis following autologous fat grafting is an exceptionally rare complication. Volume restoration of preauricular post-cancer deformities with fat grafting is commonly performed. Although several cases of facial nerve palsy have been reported after injection of synthetic dermal fillers, transient total paralysis specifically caused by autologous fat grafting in an irradiated free flap has not been documented in the literature [3,4]. In this patient, the sudden onset of House–Brackmann Grade V paralysis immediately after the second lipofilling session suggests a close temporal relationship between the procedure and the neurological deficit. Although the facial nerve was anatomically preserved during the initial radical resection for verrucous SCC, its surrounding tissue environment had been substantially altered by subsequent anterolateral thigh (ALT) flap reconstruction and 30 sessions of adjuvant radiotherapy (RT).

One plausible mechanism for this transient paralysis is acute pressure-related neuropraxia occurring within the chronically fibrotic and relatively non-compliant irradiated tissue bed. RT induces cytokine-mediated responses that may result in excessive collagen deposition, microvascular changes, and progressive tissue fibrosis [5]. In non-irradiated soft tissue, greater tissue compliance may allow accommodation of injected volume with relatively limited increases in local interstitial pressure. In contrast, fibrosis and impaired lymphatic drainage after irradiation may reduce tissue compliance and its capacity to accommodate additional volume [6]. We therefore hypothesize that the injected fat, together with procedure-related edema, may have transiently increased local tissue pressure around the facial nerve trunk, potentially resulting in a reversible pressure-related neuropraxia [7]. However, no direct measurements of tissue pressure, radiological evidence of nerve compression or edema, or objective evidence of neural ischemia were obtained in this case. Accordingly, this proposed pressure-related mechanism remains hypothetical and should not be interpreted as a demonstrated pathophysiological process.

Another possible explanation is transient local anesthetic–related nerve blockade caused by residual anesthetic associated with the fat-harvesting procedure. This possibility cannot be completely excluded. However, if local anesthetic diffusion had been the predominant mechanism, an earlier resolution corresponding to the expected pharmacological duration might have been anticipated. In the present case, severe facial weakness persisted beyond the immediate postoperative period before rapid neurological recovery occurred. Although this temporal pattern may be compatible with a transient neuropraxic process, it does not allow definitive differentiation between pressure-related neuropraxia and other reversible causes of facial nerve dysfunction.

A further important differential diagnosis is viral neuropathy, such as Bell’s Palsy (HSV-1) or Ramsay Hunt Syndrome(VZV). Surgical stress may theoretically contribute to viral reactivation [8]. However, the immediate onset of paralysis following lipofilling and complete recovery of facial nerve function within 36 h are less typical of the usual clinical course of viral facial neuropathy, in which recovery generally occurs over a substantially longer period. Nevertheless, in the absence of specific virological or electrophysiological testing, a viral etiology cannot be definitively excluded.

The rapid recovery observed after initiation of systemic methylprednisolone therapy is temporally compatible with resolution of a reversible neuropraxic process. Corticosteroid therapy may theoretically reduce procedure-related edema and thereby decrease pressure within a relatively non-compliant fibrotic tissue bed [9]. Experimental and clinical evidence indicates that compression can impair neural microcirculation and nerve conduction [10]; however, the present case does not provide direct evidence that such a mechanism occurred. however, the present case does not provide direct evidence that such a mechanism occurred. Moreover, the temporal association between corticosteroid administration and neurological recovery does not establish causality. No objective assessment of perineural edema or tissue pressure was performed, and spontaneous resolution of a transient conduction block cannot be excluded. Therefore, the clinical improvement following methylprednisolone should be interpreted cautiously and should not be considered proof either of an edema-related mechanism or of a direct therapeutic effect of corticosteroid administration.

Despite these limitations, this case highlights a potentially important consideration during secondary refinement procedures in previously irradiated free flaps. Procedures such as lipofilling are generally regarded as relatively low-risk; however, altered tissue compliance and fibrosis following radiotherapy may modify the response of reconstructed tissues to additional injected volume. When fat grafting is performed in proximity to major nerve structures within irradiated reconstructive fields, cautious injection using low pressure and small aliquots may be prudent. Close postoperative neurological observation may also facilitate early recognition of unexpected nerve dysfunction. Further clinical observations and objective evaluation would be required to determine whether pressure-related neuropraxia represents a reproducible mechanism in this setting.

## 4. Conclusion

This case demonstrates rapidly reversible facial paralysis occurring immediately after autologous fat grafting into an irradiated ALT free flap. A transient pressure-related neuropraxia associated with reduced tissue compliance represents one plausible mechanism; however, this remains hypothetical in the absence of objective evidence of nerve compression, edema, or ischemia. The rapid recovery following corticosteroid administration does not establish either the underlying mechanism or a causal therapeutic effect. Cautious, low-pressure injection and close neurological observation should be considered when performing fat grafting near major nerve structures in previously irradiated reconstructive fields.

## Data Availability

The data supporting the findings of this case report are available from the corresponding author upon reasonable request.

## References

[1] Wei F, Chan Jain V, Celik N, et al. Have we found an ideal soft-tissue flap? An experience with 672 anterolateral thigh flaps. Plast Reconstr Surg. 2002;109(7):2219–2226. doi: 10.1097/00006534-200206000-00007. 12045540

[2] Dormand E, Banwell PE, Goodacre TE. Radiotherapy and wound healing. Int Wound J. 2005;2(2):112–127. doi: 10.1111/j.1742-4801.2005.00079.x. 16722862 PMC7951225

[3] Samira N, Khan NM. Bell’s palsy following lip filler: a rare presentation – association or coincidence? Medtigo J Med. 2024;1(1):1–5. doi: 10.63096/medtigo3084115.

[4] Abdulsalam AJ, Alsairafy MA, Al-Hashel JY, et al. Bilateral facial palsy after cosmetic filler injections: is pain really the price of beauty? Rev Neurol (Paris). 2020;176(7-8):634. doi: 10.1016/j.neurol.2020.02.004. 32305142

[5] Straub JM, New J, Hamilton CD, et al. Radiation-induced fibrosis: mechanisms and implications for therapy. J Cancer Res Clin Oncol. 2015;141(11):1985–1994. doi: 10.1007/s00432-015-1974-6. 25910988 PMC4573901

[6] Rojas-Rojas L, Ulloa-Fernández A, Castro-Piedra S, et al. Evaluation of biomechanical and chemical properties of gamma-irradiated polycaprolactone microfilaments for musculoskeletal tissue engineering applications. Int J Biomater. 2022;2022:5266349. doi: 10.1155/2022/5266349. 35528848 PMC9076351

[7] Gao Y, Weng C, Wang X. Changes in nerve microcirculation following peripheral nerve compression. Neural Regen Res. 2013;8(11):1041–1047. doi: 10.3969/j.issn.1673-5374.2013.11.010. 25206398 PMC4145887

[8] Goo B, Kim J-H, Park J, et al. Recovery rate and prognostic factors of peripheral facial palsy treated with integrative medicine treatment: a retrospective study. Front Neurol. 2025;16:1525794. doi: 10.3389/fneur.2025.1525794. 40170900 PMC11958222

[9] Kim J, Moon IS, Lee WS. Effect of delayed decompression after early steroid treatment on facial function of patients with facial paralysis. Acta Otolaryngol. 2010; 130(1):179–184. doi: 10.3109/00016480902896154. 19437165

[10] Salgado Alves Vilela D, Roberto Lazarini P, Ferreira Da Silva C. Effects of hyperbaric oxygen therapy on facial nerve regeneration. Acta Otolaryngol. 2008;128(9):1048–1052. doi: 10.1080/00016480701827525. 19086199

